# Mr. Potts, *on the Means of Supplying the Loss of Amputated Limbs*

**Published:** 1801-03

**Authors:** James Potts, W. Lynn

**Affiliations:** Parliament Street


### Mr. Potts, *on the Means of supplying the Loss of amputated Limbs.*

A Patent has been obtained for a contrivance, which, in point of ingenuity, elegance, and utility, furpaffes every thing which has yet been attempted of a fimilar nature. It is known, that where amputation has been performed above the knee, any artificial leg which has yet been invented to fupply the lofs, is very imperfect, as the perfon ufing it is obliged to make a femicircular motion with it in walking, and as the genuflexion or movement at the knee is fo imperfect as to make the motion very aukward, inconvenient, and unnatural.

Mr. Potts, the patentee, whofe attention was drawn to the fubject by the lofs of his own leg, has conftructed an artificial one, which he himfelf has worn for years, and which is poffeffed of the following fuperior advantages: The knee and ancle joints are entirely at the command of the wearer; and the appearance of their motions is fo natural, as very nearly to conceal the lofs of the extremity: The leg is made of light materials, and indeed of fuch as imitate both the bony and flefhy parts. It is worn with eafe and perfect fafety; it does not injure the drefs, which other artificial legs are obferved to do.

do. The wearer can kneel and rife up; can fit down and rife up; can pull on a boot, and permit it to be drawn off by a boot-jack; he can turn the anterior part of the foot outwards and inwards; ride on horfe-back with perfect fafety, and imitate almoft every natural motion without any affiftance of his hands. The patentee, who has fuffered an amputation above the knee, can walk eight or ten miles with his leg of this conftruction without fatigue.

(Signed) JAMES POTTS.

---

To Dr. BRADLEY.

Dear Sir,

Being convinced that the above invention is far fuperior to any thing I have hitherto feen, (and I have had frequent opportunities of examining the inventions of others) I take the liberty of recommending it to the readers of your very popular Journal, and fincerely hope the ingenious inventor will meet that encouragement which his merit entitles him to expect.

I am, &c.

*Parliament Street, Feb.* 21, 1801. W. LYNN.

P. S. I have the fatisfaction to add, that I have introduced this invention to many of the principal furgeons in London, all of whom coincide in opinion with myfelf.

